# Expression of *TRPM6* and *TRPM7* in the preterm piglet heart

**DOI:** 10.3389/fped.2022.891722

**Published:** 2022-08-23

**Authors:** Elizabeth M. Forbes, Bhavisha A. Bakrania, Sarah E. Steane, Karen M. Moritz, Barbara E. Lingwood, Yvonne A. Eiby

**Affiliations:** ^1^Queensland Children’s Hospital, Brisbane, QLD, Australia; ^2^UQ Centre for Clinical Research and Perinatal Research Centre, Faculty of Medicine, The University of Queensland, Brisbane, QLD, Australia; ^3^School of Biomedical Sciences, The University of Queensland, Brisbane, QLD, Australia; ^4^Faculty of Medicine, The University of Queensland, Brisbane, QLD, Australia

**Keywords:** preterm, cardiovascular function, *TRPM7*, sex differences, cardiovascular instability

## Abstract

Preterm infants are at increased risk of death and disability, and cardiovascular instability after birth is a contributing factor. Immaturity of calcium handling in the preterm heart may limit myocardial contractility and cardiac output. Two transmembrane cation channels, TRPM6 and TRPM7, may regulate intracellular cardiac calcium in the neonatal period. The aim of this study was to determine *TRPM6* and *TRPM7* mRNA expression in piglet hearts in late gestation, and the effects of sex, maternal glucocorticoids, and the transition to extrauterine life. Left and right ventricular tissue was collected at a range of gestational ages from cesarean delivered piglets at birth and at 6 h old. Additional groups included piglets exposed to maternal glucocorticoid treatment and spontaneously born term piglets at 12–24 h old. *TRPM6* and *TRPM7* mRNA expression was measured using RT-qPCR. Males had significantly lower *TRPM7* expression in the left ventricle across all gestational ages compared to females. At term, both ventricles had higher *TRPM7* expression at 6 h old than at birth. In preterm piglets, *TRPM7* expression only increased postnatally in the right ventricle following maternal glucocorticoid exposure. At 12–24 h old, *TRPM7* expression in both ventricles was lower than levels in 6 h old term Caesar piglets (113 days). Male preterm piglets may have immature myocardial Ca^2+^ handling and this could contribute to their poorer outcomes. Increased *TRPM7* expression is the mature response to birth that is missing in preterm neonates. *TRPM7* could serve as a novel target to improve cardiac function in preterm neonates.

## Introduction

Cardiovascular instability on the first day of life in preterm infants is associated with significant morbidity and mortality (1, 2). More than 20% of extremely preterm infants die within 28 days of birth (3). Among survivors, more than 30% have neurodevelopmental impairment and 10–17% develop cerebral palsy (4, 5). Traditional approaches to supporting preterm cardiovascular function focus on improving cardiac performance using crystalloid volume expansion and/or inotropic support, usually with dopamine or dobutamine. However, Cochrane reviews indicate that neither approach is effective for improving neurodevelopmental outcomes (6, 7), and in fact mounting evidence indicates that these approaches are associated with poorer outcomes (8). Current interventions are based on adult physiology, which does not always apply to the immature preterm cardiovascular system and this may explain why these treatments are ineffective. Since there are no effective alternative options, these therapeutic approaches remain the mainstay of preterm cardiovascular support in clinical practice. Novel therapies that target the unique physiology of preterm infants are critical to the development of effective interventions to support preterm cardiovascular function.

An essential first step in developing new therapies is understanding the factors that contribute to cardiovascular instability in the preterm infant. Contractile function is dependent on efficient calcium handling. In the mature heart, calcium-induced calcium release (CICR) triggers Ca^2+^ release from the sarcoplasmic reticulum, increasing cytosolic Ca^2+^ and hence activating muscle contraction. But the preterm heart is unable to utilize sarcoplasmic reticulum calcium instead relying on calcium movement from the endoplasmic reticulum (9–11). This difference in calcium handling may limit the capacity of the preterm heart to increase contractility to meet the demands of the transition to *ex utero* life. The transient receptor potential (TRP) channels, TRPM7 and TRPM6, are magnesium- and calcium-permeable ion channels that are critical regulators of magnesium homeostasis. Thus, they may have an important role in regulating the influx of calcium across the plasma membrane and maintaining calcium levels in the endoplasmic reticulum (12). So these channels have the potential to improve cardiac function through regulation of store-operated calcium entry. TRPM7 has a critical but complex role in cardiac development and function (13, 14) so it is critical to understand how differences in gestational age at birth, sex and maternal glucocorticoids exposure influence expression of this key protein. Cardiac *TRPM7* expression has been detected in the fetal mouse as early as E9.5 (15) and is highly expressed throughout gestation with 2–4-fold higher levels than in adult mice (16). The timing of its downregulation after birth is unknown. It is possible that TRPM7 plays an important role in the increase in contractility that is necessary for the successful transition to *ex utero* life. A transient increase in the hours after birth may be necessary to support the high contractility required to transition successfully, at least until maturation of other calcium handling pathways which likely occurs over the subsequent days. TRPM7 could also mediate cardiac contractility through regulation of Mg^2+^ in the myocardium, as the Mg^2+^/Ca^2+^ ratio is important in determining the amount of Ca^2+^ available for intracellular signaling and binding (15). Surprisingly, *TRPM6* expression was also detected in the fetal mouse heart (16–18), however, it is not known whether it has an important role in the heart unrelated to that of TRPM7. Studies of cardiac *TRPM6* and *TRPM7* expression in a large animal model across late gestation and early postnatal life is a critical first step in exploring their potential role in maintaining cardiovascular stability in preterm infants.

Glucocorticoids (GC) are commonly administered to women threatening preterm birth, to promote organ maturation (19), however their direct effect on the functional maturity of the neonatal heart is not well understood. Interestingly, infants who receive maternal glucocorticoids are less likely to require inotropes to support cardiovascular function, and if they do, require smaller doses in order to maintain perfusion (20). In the isolated working piglet heart model, preterm piglets treated with maternal glucocorticoids had significantly higher aortic flow than untreated piglets (21). In fetal mice, corticosterone exposure increased the expression of *TRPM6* and *TRPM7* in the heart, with loss of the effect after 60 h (16). Given that TRPM7 and TRPM6 are highly expressed in the fetal heart, these increases induced by maternal glucocorticoid exposure may be important for heart function during the transition to *ex utero* life and account for some of the improvements observed in cardiovascular outcomes in premature infants.

We propose that TRPM6 and TRPM7 are necessary for successful adaptation to extrauterine life and that there is reduced expression in the preterm heart particularly in those at risk of worse outcomes such as males and those not exposed to maternal glucocorticoids. Thus, the aims of this study were to: (1) determine changes in cardiac *TRPM6* and *TRPM7* expression across late gestation in piglets, and whether this differs by sex; (2) determine whether exposure to the *ex utero* environment alters cardiac *TRPM6* and *TRPM7* expression in preterm and term piglets and; (3) determine whether maternal glucocorticoid treatment alters expression of cardiac *TRPM6* and *TRPM7* in preterm piglets. We hypothesize that expression of *TRPM6* and *TRPM7* will be lower in immature animals, in male piglets, and in non-glucocorticoid treated piglets.

## Materials and methods

### Animal groups and tissue collection

Tissues were collected under UQ ethics approvals (UQCCR/998/08 and UQCCR/324/12/NHMRC). Large White piglets sourced from The University of Queensland Piggery at Gatton were studied.

The following 9 groups delivered by Cesarean section were studied:

• Piglets delivered at 91 d gestation and tissue collected at birth – 91 d + 0 h.
• Piglets delivered at 91 d gestation with maternal glucocorticoid treatment and tissue collected at birth – 91 d + 0 h + GC.
• Piglets delivered at 97 d gestation and tissue collected at birth – 97 d + 0 h.
• Piglets delivered at 97 d gestation with maternal glucocorticoid treatment and tissue collected at birth – 97 d + 0 h + GC.
• Piglets delivered at 97 d gestation and tissue collected at 6 after birth – 97 d + 6 h.
• Piglets delivered at 97 d gestation with maternal glucocorticoid treatment and tissue collected 6 h after birth – 97 d + 6 h + GC.
• Piglets delivered at 100 d gestation and tissue collected at birth – 100 d + 0 h.
• Piglets delivered at 113 d gestation and tissue collected at birth – 113 d + 0 h.
• Piglets delivered at 113 d gestation and tissue collected at 6 after birth – 113 d + 6 h.

An additional group of piglets delivered spontaneously at term and tissue collected 12–24 h after birth was studied—SVD.

Maternally glucocorticoids (0.19 mg/kg body weight IM; Celestone Chronodose) were administered 48 and 24 h before delivery (denoted as + GC). The timing and per kg body weight dose is similar to that received by women presenting with threatened preterm labor. At 91 d and 97 d gestation, piglets are developmentally similar to preterm infants born at the edge of viability (21–23 week) and at 27–28 week in terms of cardiorespiratory function (22).

Details of piglet delivery has been previously published (22). Cesarean delivery was performed under anesthesia via a ventral midline incision and piglets were individually removed from the uterus. In piglets survived for 6 h anesthesia was induced with 5 mg/kg of propofol (Provive 1%; AFT Pharmaceuticals, New Zealand) administered via the umbilical vein prior to clamping the cord and immediately resuscitated. Piglets in the 0 h groups were euthasized at birth using sodium pentobarbital (0.5 mL/kg Lethabarb via umbilical vein; Virbac, Australia), and the hearts immediately excised and a complete cross-section of each ventricle was snap frozen and stored at –80°C. After all piglets had been delivered, the sow was euthanized (sodium pentobarbital; 60 mL Lethabarb IV; Virbac, Australia).

Piglets in the 6 h postnatal groups were resuscitated and maintained under standard neonatal intensive care conditions. This included administration of surfactant for preterm piglets (4 mL/kg intratracheal, Survanta; Abbvie, IL, United States). All piglets were intubated and ventilated using conventional neonatal ventilation. Animals were sedated using morphine (200 μg/kg loading dose followed by 40 μg/kg/h infusion) and midazolam (100 μg/kg loading dose followed by 120 μg/kg/h infusion). Drugs were administered along with fluids (10% glucose at 3 ml/kg/h) via 3.5 French gauge dual lumen neonatal umbilical vein catheter (Argyle, Sherwood Medical, MO, United States). As part of another study the piglets underwent a brief period of hypoxia (4% inspired oxygen for 20 min). This hypoxic stress was moderate with piglets reaching an average pH of 7.18 and arterial base excess (ABE) of -8.9 mmol/L by the end of the hypoxic period (23). This insult was designed to generate only a moderate neurohormonal cardiovascular response and occurred 2–3 h hours prior to tissue collection. At the end of the monitoring period (6–8 h of life), as for their 0 h counterparts, piglets were euthanized and the hearts immediately excised, frozen, and stored.

### Quantitative real-time reverse transcription polymerase chain reaction

RNA was extracted from cardiac tissue using Qiagen RNeasy mini RNA extraction kits. Extractions from 50 to 100 mg of frozen tissue were performed in duplicate for both the left and right ventricles of each piglet. During this process all samples were treated with DNase to remove contaminating DNA. RNA was quantified in each sample using absorbance spectrometry, and integrity was confirmed using 1% agrose gel electrophoresis.

RNA was reverse transcribed using a high capacity RNA-to-cDNA kit (Life Technologies, Applied Biosystems), using 1μg of total RNA in a volume of 20 μL. Real-time PCR was performed using custom primers and SYBR green dye. Target genes were *TRPM6* and *TRPM7*. Common housekeeping genes (e.g., β-actin) change with maturation so instead a known quantity (10^6^ copies) of Agilent Alien RNA was added to each RNA sample prior to reverse transcription to serve as an exogenous control (24). For each gene, custom primers were designed and tested. As the sequence for porcine *TRPM6* and *TRPM7* are predicted sequences only, the primers were designed based on the known sequence in human (*Trpm6* NM_017662.4; *Trpm7* NM_017672). Using the PrimerQuest tool (Integrated DNA Technologies), five candidate primers for each gene were proposed. Each candidate had an amplicon length between 60 and 200 base pairs, GC concentration of ∼50%, and a predicted melting temperature of 62°C. These candidate primers were then aligned against the predicted sequence in pigs using Primer-BLAST (NCBI, NIH) (*Trpm6* 100157775; *Trpm7* XM_003121515.4). The two candidates with the least number of mismatches were selected for each gene. Each primer forward and reverse sequence were then checked against the entire porcine genome using BLAST to ensure the sequence was specific for *TRPM6* and *TRPM7* respectively. Each primer was subject to a validation experiment to ensure the efficiency of the target and exogenous control amplifications were approximately equal (25). For each gene the primer with the best efficiency was selected (Table 1).

**TABLE 1:** Sequence of TRPM6 and TRPM7 primers.

Gene		Primer
*TRPM6*	FWD	GTGATGGATCGGGTGGATTT
	REV	CTTTCTTACCATCCCTCGACTG
*TRPM7*	FWD	GAGAGATGTGGTTGCTCCTTATC
	REV	TATTGGTGGATGATGGCACTG

Real-time PCR reactions were carried out on 2 μL cDNA (equivalent to 20 ng reverse-transcribed RNA), 1μL of forward and reverse primer, and Quantinova SYBR Green (Qiagen). Each 20 μL reaction was run in duplicate on a 96-Well QuantSudio6 Flex Real-Time PCR system (Applied Biosystems, Thermo Fisher Scientific), with H_2_O acting as a negative control. Cyclic conditions were 10 min polymerase activation at 95°C (required for Alien primer activation), and 40 cycles of 95°C for 30 s followed by 60°C for 90 s. The threshold was automatically set by the software. The ΔΔC_T_ was calculated for each sample using pooled samples from a subgroup of 113 d male and female left ventricles at 0 h as the calibrator.

To confirm the veracity of the qPCR results, the amplified products were purified using a Gel extraction kit (Qiagen Pty Ltd., Doncaster VIC, Australia) following precisely the manufacturer’s instructions. Aliquots of the purified PCR products were resolved on a 2% agarose gel and visualized by staining with 0.5 μg/mL ethidium bromide (Sigma-Aldrich, Castle Hill NSW, Australia) to check DNA quality and for estimating DNA concentration. An aliquot of the purified PCR product for each gene (1 μl, equivalent to 10 ng) together with the sequencing primer (1 μl of a 10 μM stock) was assembled in a 1.5 ml Eppendorf tube with 10 μl of MilliQ water. The DNA sample preparation was submitted to the Australian Genome Facility for BDT labeling, purification and Sanger sequencing. Chromas software was used to analyze the sequencing results. The deduced sequences were searched against the BLAST database in NCBI and confirmed that the product encoded *TRPM6* and *TRPM7* respectively.

### Statistical analysis

Statistical software program IBM SPSS v25 (SPSS Inc., Chicago, United States) was used to conduct all statistical analyses. Shapiro Wilk statistics and QQ plots were performed to assess normality of data. Expression data is presented as fold change (2^-ΔΔC_T_) and was not normally distributed. Statistical significance was set at *P* < 0.05. Litter effects were present but visualization of expression by litter using scatter plots showed that no litters had expression outside the range observed for other litters at that gestational age and so there was little evidence to suggest that further analysis without considering litters would be a flawed approach. Kruskal-Wallis and Mann-Whitney U tests were used to detect differences in mRNA expression due to the effects of gestational age, glucocorticoid exposure and postnatal age. Sample sizes are small so to test for sex differences, data across all gestational ages were combined and Mann-Whitney *U*-tests used for each gene/ventricle combination. ANOVA with Games Howell *post-hoc* test was used to detect differences in blood pressure as this parameter is normally distributed. Co-expression of *TRPM6* and *TRPM7* was assessed separately for the right and left ventricles using Spearman 2-tailed correlation analyses at each gestational age.

## Results

A total of 130 piglets were studied. The sex ratios, body and heart weights are described in Table 2. Mean arterial blood pressure was significantly lower at 97 d + 6 h + GC than their term counterparts (113 d + 6 h, *P* = 0.014).

**TABLE 2:** Characteristics of piglets across late gestation with and without exposure to the *ex utero* environment and maternal glucocorticoid treatment.

	Groups									
GA	91 d		97 d				100 d	113 d		Term SVD
PNA	0 h		0 h		6 h		0 h	0 h	6 h	24 h
GC	-	+	-	+	-	+	-	-	-	-
M:F, *n*	6:6	10:6	4:4	6:7	7:5	7:6	10:4	8:11	5:5	4:6
No. litters	3	4	4	4	4	4	2	7	4	4
BW, *g*	663 ± 168	705 ± 146	869 ± 173	1,103 ± 109	1,030 ± 218	1,098 ± 152	925 ± 249	1,364 ± 316	1,438 ± 280	1,772 ± 249
HW, *g*	4.7 ± 1.2	4.8 ± 1.0	7.0 ± 1.1	7.6 ± 1.4	7.6 ± 1.8	7.1 ± 1.1	6.5 ± 1.4	10.7 ± 2.4	10.7 ± 3.0	15.1 ± 3.3
HW:BW, *g/kg*	7.1 ± 0.8	6.9 ± 0.6	8.1 ± 1.4	6.8 ± 1.0	7.5 ± 1.2	6.5 ± 0.6	7.1 ± 0.6	6.9 ± 2.6	7.5 ± 1.3	8.4 ± 1.2
BP*	–	–	–	–	32.8 ± 7.1	31.3 ± 5.1	–	–	37.7 ± 4.2	–

GA, gestational age; PNA, postnatal age; SVD, spontaneous vaginal delivery; GC, maternal glucocorticoid treatment; BW, body weight; HW, heart weight; BP, arterial blood pressure (at 3 h after birth and prior to induced hypoxia). *Indicates that blood pressure was significantly lower in 97 d + 6 h + GC piglets compared to 113 d + 6 h piglets (P = 0.014 ANOVA). Values are mean ± SD.

### Effect of gestational age on *TRPM6* and *TRPM7* expression

**FIGURE 1:**
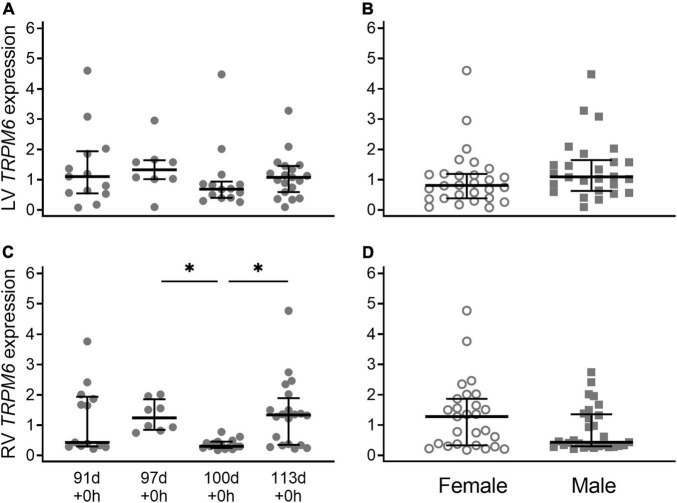
Cardiac *TRPM6* mRNA expression in the left ventricle (LV; **A,B**) and right ventricle (RV; **C,D**) of piglets during late gestation. **(A,C)** Are combined sexes; **(B,D)** are females (open circles) and males (gray squares). Plot of median (IQR) of fold change (2^-ΔΔC_T_) normalized to 113 d. *N* = 13 (91 d), 8 (97 d), 14 (100 d), 19 (113 d). *Indicates significantly lower expression at 110 d compared to 97 d and 113 d (*P* < 0.001).

Expression of *TRPM6* in the left ventricle (LV) was similar throughout gestation (*P* = 0.95) (Figure 1A). Expression of *TRPM6* in the right ventricle (RV) was bimodally distributed at 91 d and 113 d but this was not due to sex, litter, season, or inter-observer differences. Expression at 100 d was significantly lower than expression at 97 d + 0 h and 113 d + 0 h (both *P* < 0.001) (Figure 1C). There was no significant difference in expression of *TRPM6* between males and females in either the right (*P* = 0.13) or left ventricle (*P* = 0.15) (Figures 1B,D).

**FIGURE 2:**
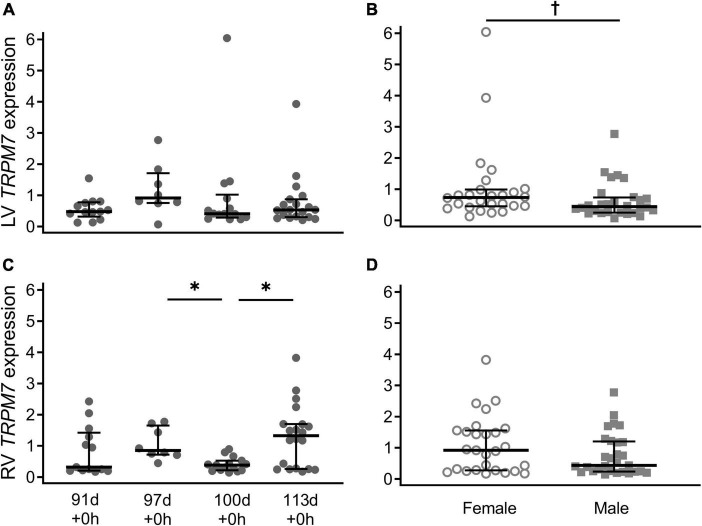
Cardiac *TRPM7* mRNA expression in the left ventricle (LV; **A,B**) and right ventricle (RV; **C,D**) of piglets during late gestation. **(A,C)** Are combined sexes; **(B,D)** are females (open circles) and males (gray squares). Plot of median (IQR) of fold change (2^-ΔΔC_T_) normalized to 113 d. *N* = 13 (91 d), 8 (97 d), 14 (100 d), 19 (113 d). *Indicates significantly lower expression at 110 d compared to 97 d and 113 d (*P* < 0.005). ^†^Indicates males at birth had lower *TRPM7* expression in the left ventricle (*P* < 0.038).

Expression of *TRPM7* in the left ventricle was similar across the gestational ages (Figure 2A; *P* = 0.55). In the right ventricle, expression of *TRPM7* was bimodally distributed and had a similar pattern to *TRPM6* expression, significantly lower at 100 d compared with 97 d + 0 h (*P* = 0.001) and 113 d + 0 h (*P* = 0.007) (Figure 2C). Male piglets had significantly lower *TRPM7* expression compared to female piglets in the left ventricle only when all gestational ages are combined (LV: *P* = 0.038, RV: *P* = 0.21) (Figures 2B,D).

### Effect of postnatal age on expression

**FIGURE 3:**
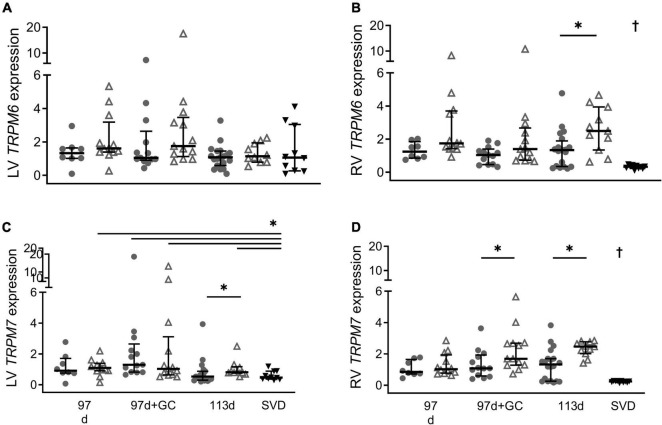
Effect of postnatal age on cardiac mRNA expression of *TRPM6* (**A:** LV is left ventricle, **B:** RV is right ventricle) and *TRPM7* (**C:** LV, **D:** RV) in piglets at birth (0 h; closed circles) and 6 h old (open triangles). Plot of median (IQR) of fold change (2^-ΔΔC_T_) normalized to 113 d + 0 h. GC is maternal glucocorticoid treatment. SVD is spontaneous vaginal delivery at term (12–24 h old; black triangles). *Indicates significant differences between groups (*P* < 0.05). ^†^Indicates expression in the right ventricle of term SVD piglets was different to all other groups. *n* = 8 (91 d), 12 (91 d + 6 h), 12–13 (97 d + GC), 13 (97 d + GC + 6 h), 19–20 (113 d), 10 (113 d + 6 h), 10 (115 d + 24 h).

Term SVD piglets had *TRPM6* expression in the left ventricle that was similar to all groups (Figure 3A, *P* > 0.09 all groups). Whereas *TRPM7* levels in the left ventricle of term SVD piglets were significantly lower than all groups (*p* < 0.05) except those without glucocorticoid treatment or postnatal exposure, i.e., 97 d + 0 h (*P* = 0.083) and 113 d + 0 h (*P* = 0.98) (Figure 3C). In the right ventricle, term SVD piglets had significantly lower *TRPM6* and *TRPM7* expression compared to any other group (Figures 3B,D, *p* < 0.004 all groups). At 6 h old, term Caesar piglets (113 d + 6 h) had higher *TRPM6* and *TRPM7* expression in the right ventricle (*P* = 0.016 and 0.013) and higher *TRPM7* expression in the left ventricle (*P* = 0.031) than their 0 h counterparts (113 d + 0 h; Figures 3B–D). This postnatal increase also occurred for *TRPM7* expression in the right ventricle of glucocorticoid exposed piglets at 97 d gestation (97 d + 0 h + GC: *P* = 0.034) (Figure 3D). Sex effects due to postnatal age were not detected (all *P* > 0.05).

### Effect of maternal glucocorticoid treatment on expression

**FIGURE 4:**
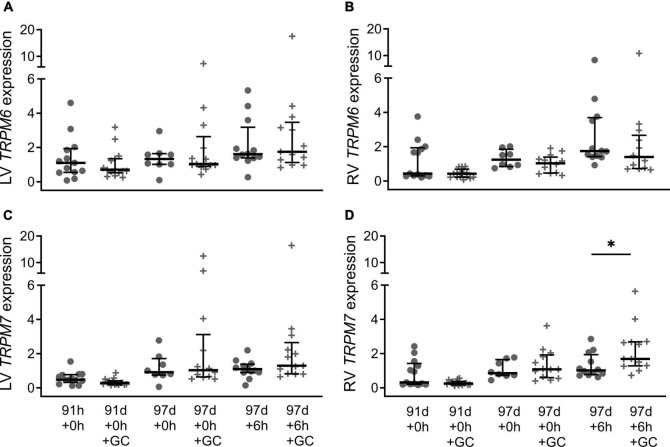
Effect of maternal glucocorticoids on cardiac mRNA expression of TRPM6 (**A:** LV is left ventricle, **B:** RV is right ventricle) and TRPM7 (**C:** LV, **D:** RV) of preterm piglets. Maternal glucocorticoid treatment (crosses) and no treatment (circles). Plot of median (IQR) of fold change (2^-ΔΔC_T_) normalized to 113 d + 0 h. *Indicates significant differences between groups (*P* < 0.05). *n* = 13–15 (91 d + 0 h), 16 (91 d + 0 h + GC), 8 (97 d + 0 h), 13 (97 d + 0 h + GC), 12 (97 d + 6 h), 13 (97 d + 6 h + GC).

Maternal glucocorticoid treatment did not have a significant effect on the expression of *TRPM6* in either ventricle at 91 d + 0, 97 d + 0 or 97 d + 6 h (Figures 4A,B; all *P* > 0.05), nor was there an effect of treatment on *TPRM7* expression in the left ventricle (Figure 4C; all *P* > 0.05). However, *TRPM7* expression in the right ventricle was higher in glucocorticoid treated piglets at 97 d + 6 h compared with their untreated counterparts at 6 h old (Figure 4D; *P* = 0.030). Sex effects due to maternal glucocorticoid exposure were not detected (all *P* > 0.05).

### Correlations between *TRPM6* and *TRPM7*

There were positive correlations between *TRPM6* and *TRPM7* expression in the left ventricle for all groups except 91 d + 0 h, 97 + 0 h and SVD, and in the right ventricle for all groups except 113 d + 6 h and SVD (Table 3).

**TABLE 3:** Correlations between *TRPM6* and *TRPM7* expression in left and right ventricles at birth.

	91 d	97 d	100 d	113 d
Left ventricle	ρ = 0.69	ρ = 0.72	ρ = 0.53	ρ = 0.71
	*P* < 0.001	*P* < 0.001	*P* = 0.049	*P* < 0.001
	*n* = 28	*n* = 21	*n* = 14	*n* = 19
Right ventricle	ρ = 0.93	ρ = 0.85	ρ = 0.83	ρ = 0.83
	*P* < 0.001	*P* < 0.001	*P* < 0.001	*P* < 0.001
	*n* = 28	*n* = 21	*n* = 15	*n* = 20

ρ is Spearman two-tailed correlation co-efficient. Data includes all 0 h animals, with or without maternal glucocorticoid treatment.

## Discussion

This study explored changes in *TRPM6* and *TRPM7* expression across gestational age, postnatal age, sex and maternal glucocorticoid exposure in piglets. We revealed a number of characteristics of *TRPM7* expression which support its role in the transition of the myocardium to extrauterine life, and therefore importance as a potential therapeutic target. The role of *TRPM6* during the transition to extrauterine life remains less clear. The key findings of this study are that (1) males had lower *TRMP7* expression in the left ventricle compared to females across all gestational ages and (2) mature piglets have a postnatal increase in *TRPM7* expression that does not occur in preterm piglets.

*TRPM7* expression is sexually dimorphic, and this could contribute to poorer outcomes in male preterm infants. Cardiac preload is low in preterm infants due to reduced blood volume and excessive vasodilation (26–28). This low preload state limits the contractile force generated by the heart. This low contractility may be further compromised if myocardial Ca^2+^ availability is reduced, leading to reduced cardiac output and tissue perfusion (21). Ca^2+^ influx through TRPM7 is essential for the maintenance of endoplasmic reticulum Ca^2+^ concentration (12), a critical source of calcium for contractile function prior to maturation of calcium handling. So upregulation of *TRPM7* may lead to increased intracellular Ca^2+^ availability and therefore increased cardiac contractility allowing the left ventricle to adapt to the increase in workload that occurs after birth (11). *TRPM7* may also increase cardiac contractility via Mg^2+^ regulation in the myocardium which alters the Mg^2+^/Ca^2+^ ratio and determines Ca^2+^ availability for intracellular signaling and binding (15). In the current study, male piglets had lower *TRMP7* expression in the left ventricle compared to females, which may result in reduced myocardial Ca^2+^ availability and therefore a reduction in myocardial contractility compared to females. If this sex difference is also present in human preterm infants, and this results in reduced contractile function, this may partly explain the poorer outcomes of male infants (29). Studies of myocardial contractility in preterm infants have not addressed sex differences in contractility (30, 31). Further pre-clinical studies could elucidate if there is a sex specific relationship between *TRPM7* expression and cardiac contractility, and whether this contributes to increased rates of morbidity and mortality in male offspring.

Upregulation of cardiac *TRPM7* is likely the mature response to birth but this does not occur in the preterm heart. At term, both ventricles have higher *TRPM7* expression at 6 h old than at birth. This upregulation after birth likely provides the necessary Ca^2+^ influx to sustain the increase in contractility required for successful transition to *ex utero* life. This is particularly true for the left ventricle which must substantially increase performance after birth, unlike the right ventricle which dominates *in utero* but has decreased overall workload after birth (32, 33). However, this increase in *TRPM7* expression after birth was not observed in the left ventricle of preterm piglets. If this is also the case in preterm infants, it may limit calcium handling such that the demand for increased contractility after birth cannot be met. Those preterm infants that also have low preload, due to excessive vasodilation and/or hypovolemia, will be particularly vulnerable to low contractility and poor cardiac performance leading to cardiovascular instability and brain injury (27).

Is maternal glucocorticoid treatment improving these outcomes in part by its influence on *TRPM7* expression? Maternal glucocorticoids are routinely administered to improve preterm outcomes. Mice without glucocorticoid receptors have reduced RNA expression of calcium handling proteins and impaired cardiac function suggesting that glucocorticoid signaling is critical for cardiac maturation (34). At 97 d gestation, glucocorticoid exposure had no effect at birth. Exogenous steroids also had no effect on fetal *TRPM7* expression at birth at a similar developmental stage in mice (ED17.5) (16). However, there is clear evidence that glucocorticoid exposure, in combination with 6 h of postnatal life, increases expression of *TRPM7* in the right ventricle of piglets, as levels are elevated at 6 h old in GC treated piglets but not in their untreated counterparts at 6 h old (Figure 4D). Whereas in the left ventricle, glucocorticoid exposure did not significantly increase expression compared to untreated piglets. If a high cardiac workload *in utero* primes the right ventricle, this could explain the difference between ventricles. In mice (E14.5), endogenous steroid exposure increased both TRPM6 and TRPM7 mRNA levels but dexamethasone, an exogenous steroid, had no effect (16). At a similar stage of development similar in piglets (91 d gestation) there was also no upregulation following administration of exogenous steroids. It’s likely that differences in the timing and type of steroid exposure during development, and its interaction with increases in cortisol and cardiac workload soon after birth, all influence changes in expression in the perinatal period. These dynamic interactions may explain the overall variability observed. Postnatal changes in *TRPM7* expression following glucocorticoid exposure suggest some of the improvement in outcomes for preterm infants following maternal glucocorticoid treatment may be due to increased TRPM7.

Upregulation of *TRPM7* expression after birth may be transient. *TRPM7* expression in the left ventricle was upregulated after birth in term piglets, but in term piglets at 12–24 h old levels were lower than at birth. This transient increase in *TRPM7* levels after birth is supported by evidence in mice where high *TRPM7* expression levels present during gestation are lost by adolescence (PN30) (16). Immediately following birth there is an increased workload, particularly in the left ventricle and this results in an increase in Ca^2+^ requirement. The transient increase in TRPM7 may support this increased requirement for intracellular Ca^2+^ prior to maturation of CICR. Downregulation in the left ventricle may occur once calcium handling has matured in the early postnatal period. If this is the case this may partly explain why in preterm infants where upregulation has not yet occurred, cardiovascular function deteriorates soon after birth. Further studies are necessary to clarify the time course and its interaction with maturation of the CICR pathway.

*TRPM6* is less ubiquitously expressed than *TRPM7* but has previously been detected in both the fetal and adult mouse heart (16). In our study, there was a bimodal distribution in the right ventricle but we were not able to identify a reason despite interrogating for effects of sex, litter, or observer. Interestingly, cardiac *TRPM6* was stably expressed throughout late gestation and across a range of experimental conditions, except for an increase at 6 h old compared to birth in the right ventricle of term piglets which decreased to lower levels by 12–24 h old. In both instances this was matched with a similar change in expression in *TPRM7*. *TRPM6* and *TRPM7* are homologs with similar permeability (35) but they are functionally non-redundant, as upregulation of *TRPM6* is unable to rescue *TRPM7* deficient phenotypes (36). TRPM7 is essential in the surface expression of TRPM6, and together they form heterotetrameric channel complexes that have unique properties compared to homomeric channels (35, 37–40). It is possible that TRPM6/7 channel complexes play a role in cardiac function separate to that of homomeric TRPM7 channels. This study also shows that cardiac expression of *TRPM6* and *TRPM7* is highly correlated at birth in preterm and term piglets, It has previously been suggested that the effect of *TRPM6* is mediated through co-expression with, or regulation of, *TRPM7* through formation of heteroterameric channel complexes (35, 37, 38) or through its regulatory action on *TRPM7* function (39, 40).

There may be potential therapeutic value of targeting TRPM7 in preterm infants particularly for males and those not receiving their full course of prenatal steroids. Two potential activators of TRPM7, naltriben and mibefradil (41), may have therapeutic benefit by increasing contractility but their clinical application in preterm infants is yet to be explored. Naltriben has been shown to activate TRPM7 channels and lead to increased intracellular Ca^2+^. These effects are rapid and fully reversible. Mibefradil is also able to stimulate TRPM7-mediated Ca^2+^ entry. A key difference between these TRPM7 agonists is that mibefradil is dependent on the intracellular concentration of Mg^2+^, in comparison to naltriben which is Mg^2+^ independent. Further exploration of the effects of these two agents in neonatal animal models are required to explore their potential in the treatment of cardiovascular compromise in early extrauterine life.

The major limitations of this study are the absence of protein expression data and assessment at limited postnatal ages. A lack of availability of a specific antibody limits the ability to perform Western blotting however, future studies of protein expression would strengthen gene expression observations within the current study. Another major limitation is that postnatal effects on expression may have been confounded by the short period of induced hypoxia. However, hypoxia is a common occurrence during the transition to *ex utero* life, and thus this hypoxia period may not represent an abnormal circumstance. Further studies elucidating the effect of hypoxia on TRPM7 are necessary to complete understanding of the role of TRPM7 in the transition to *ex utero* life. Also, piglets were only assessed at birth, 6 h and 12–24 h (term born only). It is possible that upregulation of *TRPM6* or *TRPM7* is delayed in preterm piglets compared to a term and that maternal glucocorticoids upregulate *TRPM7* in the left ventricle, but this study did not capture this later timepoint. Further studies with multiple postnatal time points are suggested to further delineate the expression of *TRPM6* and *TRPM7* in early postnatal life. Major strengths of this study include the use of a relevant model in order to draw conclusions about human neonatal physiology. It is not feasible to study tissue gene expression in human neonatal hearts, and the piglet serves as an accessible and physiologically comparable animal model. The data in this study are supported by the relatively large sample size of each treatment group. It is further strengthened by the number of gestational ages and treatment conditions that were incorporated. This allows quantification of gene expression not only across late gestation, but also during early postnatal life. The inclusion of piglets that both were or were not exposed to maternal glucocorticoids is important due to the wide use of maternal glucocorticoids clinically.

## Conclusion

This study describes the change in cardiac *TRPM6* and *TRPM7* expression during late gestation and the transition to postnatal life in the piglet and how this is altered by sex, transitioning at earlier gestational ages and in the presence of maternal glucocorticoid exposure. Upregulation of *TRPM7* is the mature response to birth but this response is largely missing in preterm piglets, particularly those not exposed to maternal glucocorticoids. Male preterm piglets have decreased *TRPM7* expression. If this results in limited calcium handling this could contribute to the poorer outcomes for male preterm infants. This suggests that TRPM7 could be a target for improving cardiac function in preterm infants.

## Data availability statement

The original contributions presented in this study are included in the article/supplementary material, further inquiries can be directed to the corresponding author.

## Ethics statement

The animal study was reviewed and approved by The University of Queensland Animal Ethics Committee.

## Author contributions

EF and YE designed the study, analyzed the data, interpreted the results, and prepared the manuscript. BB interpreted the results and prepared the manuscript for submission. SS interpreted the results and prepared the manuscript. KM designed the study and interpreted the results. BL designed the study, interpreted the results, and prepared the manuscript. All authors revised the final manuscript and approved the submitted version.
